# Mood Variability Among Early Adolescents in Times of Social Constraints: A Daily Diary Study During the COVID-19 Pandemic

**DOI:** 10.3389/fpsyg.2021.722494

**Published:** 2021-08-24

**Authors:** J. Susanne Asscheman, Kiki Zanolie, Anika Bexkens, Marieke G. N. Bos

**Affiliations:** ^1^Developmental and Educational Psychology, Faculty of Behavioral and Social Sciences, Leiden University, Leiden, Netherlands; ^2^Leiden Institute for Brain and Cognition, Leiden, Netherlands; ^3^GGZ Delfland, Department of Psychiatry in Individuals With Intellectual Disability, Center for Psychiatry, Delft, Netherlands

**Keywords:** COVID-19, adolescence, mood variability, attachment, social relationships

## Abstract

The COVID-19 pandemic and associated governmental regulations have drastically changed the daily social lives of children, adolescents, and adults. Changes in the social context may particularly affect children who are in the transition to adolescents (henceforth referred to as early adolescents) as adolescence is a crucial period for peer interactions and development of independence and autonomy. Yet, the impact of the pandemic and associated governmental regulations on early adolescents' emotional well-being has yet to be clarified. In the current study, we explored daily fluctuations in mood in 54 early adolescents (*M*_age_ = 11.07) during the first few months (April 2020–June 2020) of the COVID-19 pandemic. Moreover, the role of parents and peers on adolescents' mood variability was investigated. Adolescents rated their mood (i.e., happiness, anger, sadness, anxiety) and peer interactions once a day during four separate weeks across different weeks of containment measures in the Netherlands. Moreover, adolescents reported on their experienced attachment to parents and peers and internalizing problems during baseline and the final measurement, respectively. Results showed relatively stable levels of mood during the first few months of the COVID-19 pandemic. However, individual differences in mood variability during the first assessment week were negatively associated with the experienced level of attachment to both parents and peers. Moreover, heightened levels of mood variability did not mediate the link between attachment and internalizing problems. Lastly, the quality of offline contact, but not online contact, was negatively related to adolescents' mood variability. Overall, this study suggests that mood of early adolescents did not heavily fluctuated across the first few months of the COVID-19 pandemic. Our findings add to the growing body of literature aiming to understand how adolescent's life are affected by the COVID-19 crisis and illustrates that social connectedness to parents or peers may facilitate resilience to distress and daily mood fluctuation in early adolescents.

## Introduction

The outbreak of the corona virus (COVID-19) has led to a worldwide pandemic crisis in 2020/2021 with high mortality rates across the globe (World Health Organization, 2021). As such, many countries took drastic measures to prevent further spread of the virus. In the Netherlands, these measures included, amongst others, social distancing (i.e., keeping 1.5 m physical distance from others), working from home and closure of schools, restaurants, cinemas, and sport clubs (The Dutch Government, 2020). These social containment measures have a large impact on people's social life as they enforce proximity of families and may limit possibilities for peer interactions (Fegert et al., 2020). These changes in social context may particularly affect the emotional well-being of children transitioning into adolescents (henceforth referred to as early adolescents) as they strive to gain independence and autonomy and start spending increasingly more time with peers (Steinberg and Morris, 2001; Orben et al., 2020). Emotional well-being can be defined as individuals' tendency to experience life positively, to cope effectively with stress, and generate positive feelings and emotions (Stewart-Brown, 1998; Oberle, 2018). The aim of the current study is to examine the impact of the first few months of the COVID-19 pandemic on the emotional well-being of early adolescents, by examining adolescents' mood variability over four separate weeks of social containment measures (April–June 2020) and its associations with both parent and peer relationships.

The transition from childhood to adolescence, generally occurring between the ages of 9–12 and starting with the onset of puberty, is an emotional turbulent period characterized by increases in mood variability (Maciejewski et al., 2015). High mood variability, reflected in large and frequent changes in daily mood levels, may indicate a transition from adaptive to maladaptive mental well-being (Houben et al., 2015). As such, adolescents who show persistent high levels of mood variability may be more vulnerable to the development of internalizing problems (Maciejewski et al., 2014). Across adolescence, there is a substantial increase in the prevalence of internalizing problems (Paus et al., 2008; Solmi et al., 2021). An unanswered question is whether the COVID-19 pandemic magnifies these numbers. Even though adolescence is a period of vulnerability for emotional and mental well-being, most adolescents navigate smoothly through this developmental period. Recently, a shift in perspective has been proposed suggesting that this developmental period needs to be seen in light of social-affective opportunities, such as greater flexibility in adjusting intrinsic motivations (Crone and Dahl, 2012).

Mood levels are known to fluctuate in response to stressful events (Kuppens, 2015). The COVID-19 pandemic and subsequent governmental measures are uncontrollable stressors which are likely to have an impact on emotional well-being. However, the impact of the pandemic on emotional well-being may relate to the containment measures in place. These containment measures depend on the progression of the COVID-19 pandemic (Fegert et al., 2020). Specifically, acute phases of the pandemic required measures to reduce the spread of the virus such as social distancing and lock-downs. Following the moment the curve reached the highest numbers of new infections and the number of infections started to decline, governments were able to slowly ease regulations to return to normality, including school re-openings (i.e., recovery phase). Different phases and regulations might relate to different psychological stressors such as re-organization of family life (acute phase), social isolation (acute phase), fear of death of relatives (acute and recovery phase), economic and academic concerns (recovery phase) (Fegert et al., 2020). Recent studies showed that the COVID-19 pandemic and the associated psychological stressors indeed resulted in an increase in worry, loneliness, anxiety, and depressive symptoms in children and adults during the lock-down (acute phase) and thereafter (recovery phase) (De Quervain et al., 2020; Li et al., 2020; Orgilés et al., 2020; Whittle et al., 2020; Barendse et al., 2021; Luijten et al., 2021). Yet other studies showed no effect, or only modest effects, of the first lock-down on mood, anxiety, and depressive symptoms in children and college students (Achterberg et al., 2021; Fried et al., 2021). These seemingly contradicting findings might be due to different psychological stressors associated with different phases of the pandemic that may have an impact on early adolescents' mood and emotional well-being (Mcmahon et al., 2020). Yet, our knowledge on the impact of the still ongoing COVID-19 pandemic on early adolescents' mood variability and emotional well-being has still to be further clarified. A detailed understanding of how early adolescents experience and adapt to the COVID-19 pandemic is essential to provide support (e.g., by school and prevention programs or intervention programs) to early adolescents in emotional need, specifically given that the COVID-19 pandemic is still ongoing.

Daily fluctuations in early adolescents mood (i.e., mood variability) during the pandemic may depend on the amount of emotional and social support adolescents receive from parents and peers (Laible et al., 2000; Wilkinson, 2004). Previous research using ecological momentary assessment methods showed that high levels of parental and peer support reduced negative affect among adolescents (Weinstein et al., 2006; Bai et al., 2017; Janssen et al., 2020). Likewise, adolescents who have close parent or peer relationships or a secure attachment to their parents or peers reported fewer internalizing problems compared to adolescents who reported a more conflictual peer relationship or insecure attachment to their parents or peers (Laible et al., 2004; Dujardin et al., 2016; Gorrese, 2016; He et al., 2018). As such, high levels of attachment to parents and peers may mitigate the impact on early adolescents' mood variability during the COVID-19 pandemic. Indeed, recent research on the impact of the COVID-19 pandemic on emotional well-being of children and adolescents showed that having a good relationship with parents and peers may buffer some of the psychological stress during the COVID-19 pandemic (Whittle et al., 2020; Achterberg et al., 2021). Here, we examined whether the expected relation between parent and peer attachment with internalizing problems was mediated by mood variability. Moreover, we explored the effects of the containment measures on the quantity and quality of the social interactions both in real-life and online with peers and its relation to mood variability in early adolescents.

**Figure 1:**
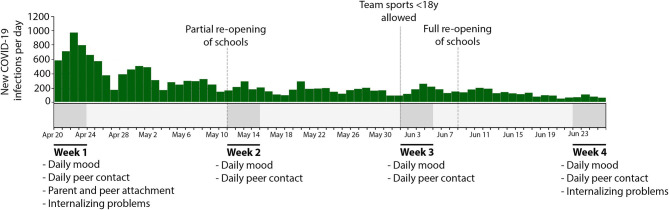
Overview of assessment weeks and daily new registered COVID-19 infections and containment related events during the study period from April to June 2020.

In the current study we examined changes in early adolescents' mood variability during the first few months of the COVID-19 pandemic and investigated potential links with parent and peer attachment and internalizing problems. The current study protocol, hypotheses and analyses plan were preregistered on June 18, 2020 on the Open Science Framework (see our OSF-page: https://osf.io/3fqky/). Participants, early adolescents aged 9–12 years, rated their mood (i.e., happiness, anger, sadness, anxiety) and peer interactions once a day (Monday–Friday) during four separate weeks across different weeks of containment measures in the Netherlands (see Figure 1). Adolescents also reported on their experienced attachment to peers and parents at baseline and on their internalizing problems at baseline and during the final assessment. It was hypothesized that the COVID-19 pandemic would initially lead to high levels of mood variability but would gradually decline when containment measures were eased. In addition, we hypothesized a relation between mood variability and both peer and parent attachment. It was expected that lower levels of self-reported attachment to parents and peers would be associated with higher mood variability. Internalizing problems were assessed to examine emotional well-being during the pandemic. We hypothesized a relation between parent/peer attachment and internalizing problems (Neumann et al., 2011; Maciejewski et al., 2014), and we expect this relation to be mediated by mood variability. Last, we exploratively assessed the effect of the containment measures on the quantity and quality of offline and online social interactions with peers and how this relates to early adolescents' mood variability.

## Materials and Methods

### Participants

In total, 54 adolescents participated in this study (59.3% girls, *M*_age_ = 11.07 years, *SD* = 0.77, range = 9.40–12.39 years). Participants were recruited from an ongoing longitudinal study (“BrightWave”) aimed to examine the role of puberty on the social-emotional development of (gifted and typically intelligent) early adolescents between ages 9–12 years. Parents of 132 adolescents were approached by email to participate in the current daily diary study. Of these 132, 62 parents provided informed consent for their child. Adolescents with informed consent did not differ in age [*t*_(130)_ = 0.11, *p* = 0.91], gender [χ${}_{\left(1\right)}^{2}$ = 2.48, *p* = 0.12], and type of education [gifted vs. non-gifted; χ${}_{\left(1\right)}^{2}$ = 0.79, *p* = 0.37] from adolescents without consent. Eight participants were excluded for further analyses; two participants never completed any assessment, and six participants did not provide enough valid data to calculate mood variability on any of the assessment weeks (see Mood variability). Hence, the final sample consisted of 54 adolescents. Importantly, adolescents attending education for gifted (*n* = 19) and typically developing children (*n* = 35) did not significantly differ in any of our study variables (i.e., mood variability, attachment, or internalizing problems) or in sex distribution. Hence, education type was not entered as covariate in our analyses. Participants were from a Dutch (64.5%), mixed (11.3%), Turkish (1.6%), or Russian ethnic background (1.6%) and information on ethnic background of 8.1% of the participants was missing. The median annual household income was above €76.000, which is 1.8 times higher than the average household income of €41.000 in the Netherlands (Lau et al., 2011).

### Procedure

The study was conducted online. Participants completed 4 weeks of online daily dairies separated by a 2-week interval between April 2020–June 2020 (see Figure 1). These weeks covered different phases of containment measures: from March 15, schools, restaurants, sport clubs and gyms closed and everyone was asked to stay home as much as possible and keep 1.5 m distance from others in public spaces. Children up to 13 years old were still allowed to play with each other with <1.5 m distance if supervised by an adult. The supervising adults were obligated to keep 1.5 m distance. On May 11, primary schools were partly re-opened, with many schools providing only half days or every other day in class education. On June 1, restaurants re-opened their doors and children and adolescents under 18 were allowed to perform sports again without adhering to the 1.5 m distance rule. On June 8, schools were fully re-opened.

During each assessment week, participants received an e-mail invitation daily at 4:30 p.m. on five consecutive weekdays (Monday–Friday) with a link to the questionnaires. Participants were reminded to fill out the questionnaire by a reminder e-mail sent at 7:00 p.m. and 8:30 p.m., if no response was received by that time. Participants completed questions daily on their mood and social and physical activities. Moreover, on the first day (April 20) participants completed an extended questionnaire including questions about internalizing problems, parent attachment, and peer attachment. On the last day of the study, participants again completed questionnaires on internalizing problems. A total of 929 daily diaries were completed on time (i.e., completed on the afternoon/evening due or the next morning before 11.59 a.m.).

A detailed overview of all the used measures can be found on our OSF-page (https://osf.io/3fqky/). Note that not all measurements were reported in the current paper. Participants received a maximum of €12 with €2 euros for completing the start measure, €2 euros for the end measure and €0.40 euros for each completed daily assessment (range of final payments was €2.50–€12). All study procedures were approved by the ethical review board of Psychology at Leiden University (CEPnr. 2354).

### Measures

#### Mood Variability

Each day participants rated the intensity of their mood over the day on four different mood scales using the Daily Mood Device, an internet version of the Electronic Mood Device (Hoeksma et al., 2000). Each scale consisted of three items, assessing the participants mood over the day (i.e., *Today, I feel…*). The four different mood scales were happiness (i.e., glad, happy, cheerful), anger (i.e., angry, cross, short-tempered), sadness (i.e., sad, down, dreary), and anxiety (i.e., afraid, anxious, worried). Participants answered these 12 mood items on a 9-point Likert scale with 1 indicating not happy/sad/etc. and 9 indicating very happy/sad/etc. To determine the between-persons reliability of the scales, we calculated the R1F which reflects the reliability of the scale items to separate individuals from each other on a single fixed day with values above 0.70 indicating acceptable reliability (Cranford et al., 2006). The R1F value for happiness was 0.80, for anger 0.76, for sadness 0.83, and for anxiety 0.59. The omega coefficient, rather than Cronbach's alpha, was used to determine the within-person reliability for each scale separately as omega has been shown to outperform Cronbach's alpha (Mcneish, 2018). Due to the nested nature of the data (i.e., days within weeks within persons), a three-level confirmatory factor analysis was specified to assess reliable within person changes over time, with the items per scale on the first level, the different weeks on the second level, and ID number on the third level. The omega coefficient for happiness was 0.72, for anger 0.74, for sadness 0.79, and for anxiety 0.60. Thus, the scales showed high within-person reliability.

To assess mood variability, a weekly mood variability score was calculated for each mood scale separately using the mean absolute difference score (average mood levels are reported in Supplementary Table 1 for completeness). Hence, data was only valid when participants completed the Daily Mood Device on at least 4 days or three consecutive days in 1 week (Monday–Friday). The absolute difference between scores on consecutive days was summed and divided by the number of assessments in that week (i.e., to account for missing data). As such, the mood variability score per week captures the temporal ordering of the emotion and the change in intensity from day-to-day.

We decided to compute the average of all four mood scales to obtain an overall mood variability scores as prior studies showed that the different mood variability scales were highly correlated and loaded on one factor per assessment week (Maciejewski et al., 2019). Moreover, prior studies showed that the different mood variability scales contribute to internalizing problems in a similar manner (Silk et al., 2003; Neumann et al., 2011). Results of an exploratory factor analysis (Supplementary Table 2) confirmed that the different mood variability scales loaded on one factor in our study, with the exception of week 1 (Factor 1: sadness and anger; Factor 2: anxiety and happiness). Hence, we used one overall mood variability score per week (factor loadings: 0.49–0.87; explained variance: 47.4–60.8%). As such, in our analyses we used weekly mood variability scores for the four assessment weeks per participant to assess within-person changes in mood variability during the COVID-19 pandemic.

#### Parent and Peer Attachment

A shortened version of the Inventory of Parent and Peer Attachment (IPPA) (Raja et al., 1992) was used to measure perceived attachment to peers and parents. The questionnaire consisted of 12 items on three subscales (communication, trust, alienation) of four items each for mother, father, and peers separately. The communication subscale assesses the perceived quality of communication with parents or peers (e.g., “*My friends encourage me to talk about my problems*”). The trust subscale assesses participants perception of mutual trust in their parent or peer relationship (e.g., “*My friends listen to what I have to say*”). The alienation subscale assesses to what extent participants experience anger or alienation in their relationship with their parents or peers (e.g., “*I feel alone when I am with my friends*”). Items can be answered on a 5-point Likert scale ranging from 0 (Never or Almost never) to 4 (Always or Almost always). Negative items were reverse coded, and a mean score was calculated for mother, father, and peer attachment. As we expected that high attachment to either mother or father can act as protective factor for behavioral and emotional problems (Duchesne and Ratelle, 2014; Kochanska and Kim, 2014), the highest score for either mother or father was used as a measure of parent attachment (68.5% mother, 22.2% father, 9.3% both). Higher scores on parent attachment or peer attachment indicated higher perceived attachment to parents and peers. The omega coefficients of the parent and peer attachment scale (mother_ω_ = 0.70; father_ω_ = 0.80; peer_ω_ = 0.87) indicated good reliability and was similar to other studies (e.g., Udell et al., 2010; Asscheman et al., 2020). The omega coefficient (ω) of the individual subscales can be found in Supplementary Table 3 and ranged between ω = 0.34–0.84.

#### Internalizing Problems

The Strengths and Difficulties Questionnaire (SDQ) was used to assess internalizing problems (Goodman, 2001; Muris et al., 2003). The SDQ consists of 25 items on the behavior (emotional symptoms, conduct problems, hyperactivity/inattention, peer problems, prosocial behavior) of the participant during the past 6 months. Items could be answered on a 3-point Likert scale with 0 = not true, 1 = somewhat true and 2 = certainly true. Internalizing problems were assessed with the subscales emotional problems (5 items; e.g., “*I worry a lot*”) and peer problems (5 items; e.g., “*I have one good friend or more*”). Positive items were reverse coded and a mean score of internalizing problems was computed with higher scores indicating more internalizing problems. The omega coefficient indicated good reliability (omega = 0.75).

#### Time Spent With Peers

Participants were asked daily to indicate how many minutes they spent with their peers face-to-face (henceforth referred to as offline) and online (e.g., WhatsApp, gaming) that day. For offline contact, the following open-ended question was presented: “*How many minutes did you meet with friends or with children from your neighborhood?*”. For online contact, the following open-ended question was presented: “*How many minutes did you talk to your friends by calling/WhatsApp/social media/gaming?*”. Moreover, participants were asked to rate how much they enjoyed the offline and online contact on a scale from 1 to 10 as a measure of the experienced quality of the contact. We calculated the average time spent with peers and the average enjoyability per assessment week separately for offline and online contact. Higher averages indicated more time spent with peers and higher experienced enjoyability of the contact.

### Analyses

Our analysis plan was 4-fold (see OSF-page: https://osf.io/3fqky/). First, we assessed the trajectory of mood variability. Second, we examined the association between parent and peer attachment and mood variability. Third, we examined whether mood variability mediated links between parent and peer attachment and internalizing symptoms. Fourth, we explored the time and quality of offline and online contact with peers and examined how this was related to mood variability. Results were considered significant at *p* <0.05 (Bender and Lange, 2001). Our analysis scripts are available on our OSF-page (https://osf.io/3fqky/) and data is available at dataverse (link can be found on our OSF-page https://osf.io/3fqky/).

#### Mood Variability Trajectory

To examine adolescents' trajectory of mood variability during the COVID-19 pandemic, latent growth curve models (LGM) were fitted using the Lavaan package (Rosseel, 2012) in R (R Core Team, 2018). In our LGM, latent factors were estimated for both the intercept and slope of mood variability to determine the course of mood variability during the first few months of the COVID-19 pandemic. The intercept parameter refers to the estimated starting point of the curve at the centered time point of mood variability. In the present study the intercept was centered at the first assessment week. The slope parameter refers to the change in mood variability over time. We fitted a linear and quadratic model to determine which slope captured the trajectory of mood variability better. The Little's MCAR test showed that the data was missing completely at random (test on all study variables: χ^2^ = 54.20, *df* = 70, *p* = 0.918). Therefore, maximum likelihood estimation with robust standard errors (MLR) was used to account for the missingness and non-normality of the data. Model fit was considered acceptable if the Comparative Fit Index was above >0.95 (CFI) (Bentler, 1990) and the Root Mean Square Error of Approximation (RMSEA) (Browne and Cudeck, 1992) and Standardized Root Mean Square Residual (SRMR) (Bollen and Curran, 2006) were below <0.08. The linear model was formally compared to the quadratic model using the Satorra-Bentler χ^2^ difference test (Satorra, 2000). Sex (dummy coded: 1 = male and 2 = female) was added to the best fitting growth curve to control for potential confounding effects of sex on the intercept and slope of mood variability.

#### Relation Between Parent and Peer Attachment and Mood Variability

We also examined the relation between parent attachment, peer attachment, and mood variability. The individual intercept and slope parameters per participant from the best fitting latent growth curve model were extracted. These individual intercept and slope parameters were used as dependent variables in the hierarchical regression analyses using SPSS version 26.0. Data inspection showed that the residuals of the overall parent and peer attachment scale as well as the different attachment subscales (alienation, trust, communication) were not normally distributed. Therefore, a square transformation was used for the overall attachment scale as well as the alienation, trust, and communication subscales. For the alienation subscale, a square root transformation was used. Further statistical analyses with parent or peer attachment were performed with the transformed data.

We first regressed attachment on the individual intercept and slope parameters of mood variability. As parent and peer attachment were moderately correlated (*r* = 0.51), two separate regression analyses were performed, namely one for parent and one for peer attachment, to examine the independent effects of parent and peer attachment on mood variability. To these regression models, sex was added in a second step as covariate. Additionally, we explored the association between intercept and slope parameters of mood variability on the individual attachment subscales (alienation, trust, communication).

#### Mood Variability as Mediator Between Attachment and Internalizing Problems

In the third part of our analysis, we examined whether the association between attachment to parents or peers (assessed at baseline, week 1) and internalizing problems on the last assessment was mediated by mood variability (i.e., individual intercept and slope parameters). To this end, mediation analyses were performed using the PROCESS macro (model 4) in SPSS (Preacher and Hayes, 2008). Significance of the indirect effects were determined using bootstrapping with 10,000 samples and a 95% confidence interval. Sex was added as covariate.

#### Time Spent With Peers

Last, we explored how the average time and grade of peer contact per week was correlated with the mood variability score per week to gain insight into potential associations between mood variability and time or quality of the peer contact. As prior studies suggested potential sex differences in the duration and frequency of peer interactions, we performed additional analysis in which we controlled for sex when examining the correlation between time and grade of peer interaction and mood variability (Rose and Rudolph, 2006).

## Results

Descriptive statistics and correlations of mood variability, parent attachment, peer attachment, and internalizing problems are shown in Table 1.

**Table 1:** Correlations and descriptive statistics of study variables.

	**1**	**2**	**3**	**4**	**5**	**6**	**7**	**8**	**9**	**10**	**11**	**12**	**13**	**14**	**15**	**16**	***M***	***SD***	**Range**
1. Mood variability T1	–																1.34	0.81	0–3.65
2. Mood variability T2	**0.34**	–															1.33	1.34	0–7.10
3. Mood variability T3	**0.40**	**0.49**	–														1.23	1.06	0–4.20
4. Mood variability T4	0.16	0.25	**0.41**	–													1.48	1.06	0–3.35
5. Mood variability Intercept	**0.98**	**0.50**	**0.54**	0.25	–												1.32	1.06	0.36–2.86
6. Mood variability slope	**−0.63**	0.05	0.32	**0.57**	**−0.51**	–											0.02	0.18	−0.54–0.60
7. Parent attachment T1	−0.27	−0.18	−0.17	−0.30	−0.27	0.03	–										3.36	0.44	1.92–4.00
8. Parent alienation T1	**0.32**	0.24	0.18	**0.34**	**0.30**	−0.02	**−0.81**	–									1.09	0.67	0–2.75
9. Parent communication T1	−0.22	0.02	−0.22	−0.13	−0.19	0.01	**0.84**	**−0.55**	–								3.27	0.59	1.75–4.00
10. Parent trust T1	−0.15	−0.03	−0.10	−0.27	−0.13	−0.01	**0.70**	**−0.44**	**0.45**	–							3.75	0.35	2.75–4.00
11. Peer attachment T1	−0.17	−0.22	−0.28	−0.30	−0.20	−0.15	**0.51**	**−0.36**	**0.44**	**0.43**	–						2.84	0.68	0.25–3.91
12. Peer alienation T1	0.24	0.12	0.21	**0.36**	0.26	0.08	**−0.52**	**0.49**	**−0.37**	–**0.36**	**−0.80**	–					0.79	0.78	0–3.25
13. Peer communication T1	−0.06	−0.19	−0.26	−0.09	−0.09	−0.14	**0.28**	−0.04	**0.34**	**0.30**	**0.78**	**−0.32**	–				2.08	0.90	0–4.00
14. Peer trust T1	−0.12	−0.22	−0.20	**−0.34**	−0.16	−0.16	**0.49**	**−0.40**	**0.39**	**0.41**	**0.91**	**−0.72**	**0.57**	–			3.23	0.77	0–4.00
15. Internalizing T1	**0.32**	0.07	0.25	**0.35**	**0.32**	−0.03	**−0.34**	**0.50**	−0.14	−0.23	**−0.53**	**0.67**	−0.16	**−0.53**	–		0.36	0.29	0–1.30
16. Internalizing T4	0.22	0.08	0.24	**0.46**	0.23	0.17	−0.17	**0.37**	0.07	−0.26	**−0.41**	**0.58**	−0.09	**−0.40**	**0.72**	–	0.35	0.31	0–1.40

*Both parent and peer attachment measures were only assessed at T1. Bold correlation coefficients are significant at p <0.05. Possible range mood variability = 0–9, parent or peer attachment scales = 0–4, internalizing = 0–2. T1–T4 = Week 1–Week 4*.

### Mood Variability Trajectory

**Figure 2:**
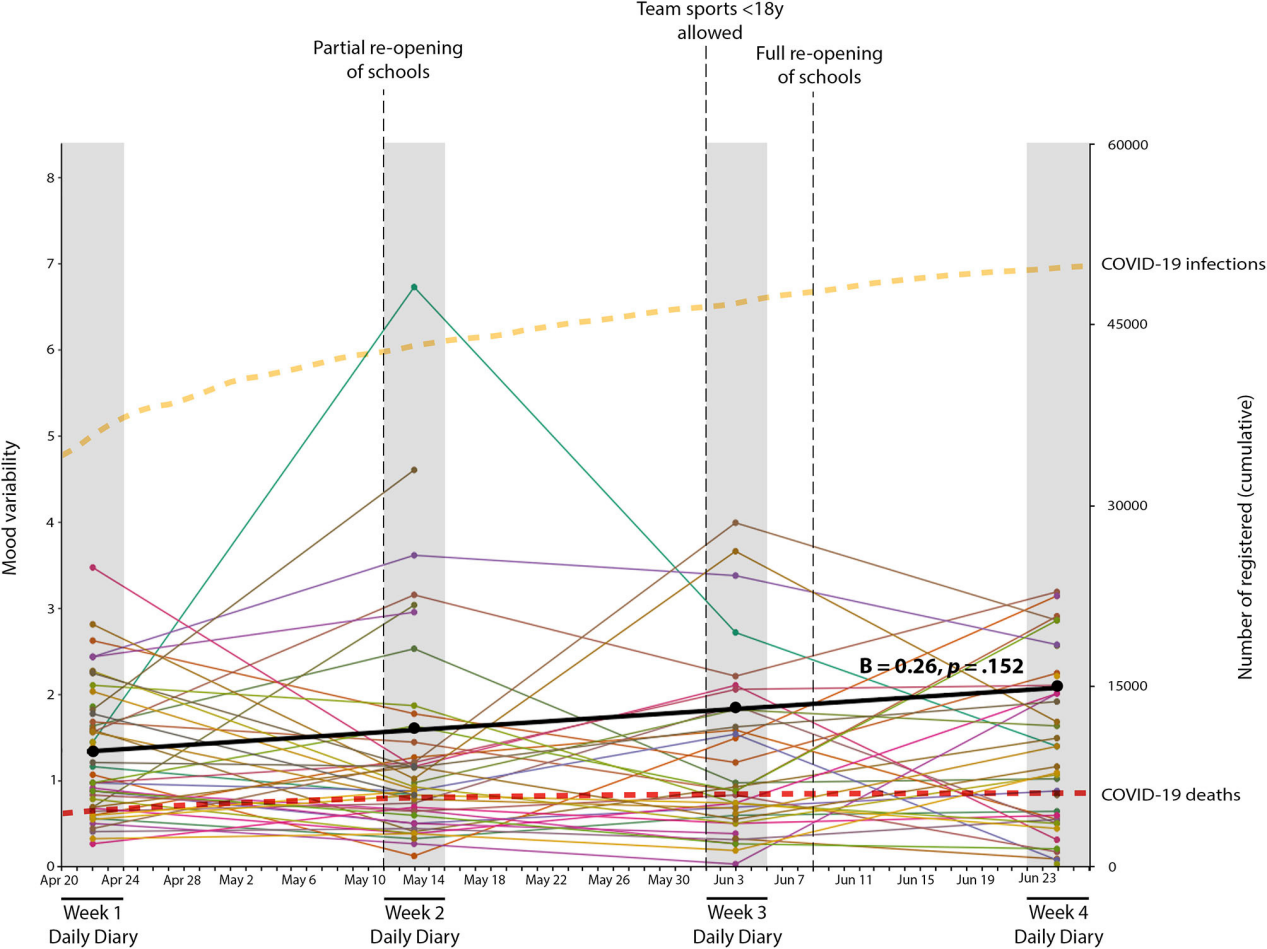
Mean mood variability trajectory and individual trajectories during the first few months of the COVID-19 pandemic. The black line represents the optimal fitting model. Colored lines represent individual participants.

Our first aim was to examine the trajectory of mood variability over the period from April to June 2020. Our LGM analyses showed that compared to the model with the quadratic slope, the model with a linear slope fitted the data better (Δχ^2^ = 3.68, Δ*df* = 4, *p* = 0.45) and had an acceptable model fit (CFI = 1.00; RMSEA = 0.00; SRMR = 0.079). Therefore, sex was added as covariate to this linear model. Model fit parameters of this final model showed that the intercept was significantly different from zero (*B* = 1.13, *SE* = 0.4. *p* = 0.005) and showed significant variance (*B* = 0.45, *SE* = 0.14, *p* = 0.002). This suggests that early adolescents' mood showed significant variability during the first assessment week but also that early adolescents differ from each other in their level of mood variability. Furthermore, the linear growth parameter (i.e., slope) was not significant (*B* = 0.26, *SE* = 0.18, *p* = 0.149) and did not show significant variance (*B* = 0.07, *SE* = 0.05, *p* = 0.152). This suggests that early adolescents' mood variability did not significantly increase or decrease during the first months of the COVID-19 pandemic covering April–June 2020 (Figure 2).

### Relation Between Attachment With Parents and Peers and Mood Variability

Our second aim was to examine whether early adolescents who experienced high attachment to parents or peers show lower mood variability during the COVID-19 pandemic. We regressed attachment to parents or peers on the extracted individual intercepts and slopes of the LGM of mood variability. As shown in Table 2, we found that adolescents' intercept of mood variability was significantly associated with parent attachment (*B* = −0.06, *SE* = 0.03, *p* = 0.036, f^2^ = 0.10) and significantly associated with peer attachment (*B* = −0.05, *SE* = 0.02, *p* = 0.04, f^2^ = 0.10) after controlling for sex. Thus, early adolescents who felt less attached to their parents or peers experienced higher levels of mood variability during the first assessment week. No significant associations were found between parent or peer attachment and the individual *slopes* of mood variability (Supplementary Table 4).

**Table 2:** Regression analyses of parent and peer attachment scales on the intercept parameters of mood variability.

	**Overall**	**Alienation**	**Communication**	**Trust**
	***B (SE)***	***B (SE)***	***B (SE)***	***B (SE)***
**Parent attachment**
*Step 1*
Constant	1.97 (0.32)^***^	0.88 (0.22)^***^	1.67 (0.24)^***^	1.76 (0.45)^***^
Attachment	−0.06 (0.03)^*^	0.45 (0.21)^*^	−0.03 (0.02)	−0.03 (0.03)
*Step 2*
Constant	1.77 (0.39)^***^	0.58 (0.35)	1.45 (0.32)^***^	1.56 (0.54)^**^
Attachment	−0.06 (0.03)^*^	0.48 (0.21)^*^	−0.04 (0.02)	−0.03 (0.03)
Sex	0.14 (0.15)	0.16 (0.15)	0.17 (0.16)	0.10 (0.16)
**Peer attachment**
*Step 1*
Constant	1.67 (0.21)^***^	1.04 (0.13)^***^	1.37 (0.13)^***^	1.66 (0.23)^***^
Attachment	−0.04 (0.02)	0.37 (0.15)^*^	−0.01 (0.02)	−0.03 (0.02)
*Step 2*
Constant	1.41 (0.29)^***^	0.77 (0.28)^**^	1.16 (0.27)^***^	1.43 (0.31)^***^
Attachment	−0.05 (0.02)^*^	0.39 (0.15)^*^	−0.02 (0.02)	−0.04 (0.02)
Sex	0.20 (0.16)	0.16 (0.15)	0.15 (0.16)	0.18 (0.16)

*Analyses were performed with the transformed data. For the overall attachment, communication and trust subscale a square transformation and for the alienation subscale a square root transformation was used.*

* *p < 0.05,*

** *p < 0. 01,*

*** *p < 0.001.*

† *p ≤ 0.10*.

For a more in depth understanding, we also explored the associations between intercepts and slopes of mood variability with the individual subscales of parent and peer attachment. We found that specifically the subscale alienation for both parents (*B* = 0.48, *SE* = 0.21, *p* = 0.028, f^2^ = 0.11) and peers (*B* = 0.39, *SE* = 0.15, *p* = 0.011, f^2^ = 0.15) was associated with mood variability during the first assessment week. No significant results were found for the subscales trust and communication (Table 2). Moreover, no associations were found when the subscales of parent and peer attachment were regressed on the individual *slopes* of mood variability (Supplementary Table 4). Together, these results suggest that adolescents who experience lower levels of attachment and specifically more alienation, irrespective of the source of attachment (parents or peers), experience higher levels of mood variability on the first assessment week assessed during the COVID-19 pandemic.

### Mood Variability as Mediator Between Peer Attachment and Internalizing Problems

**Figure 3:**
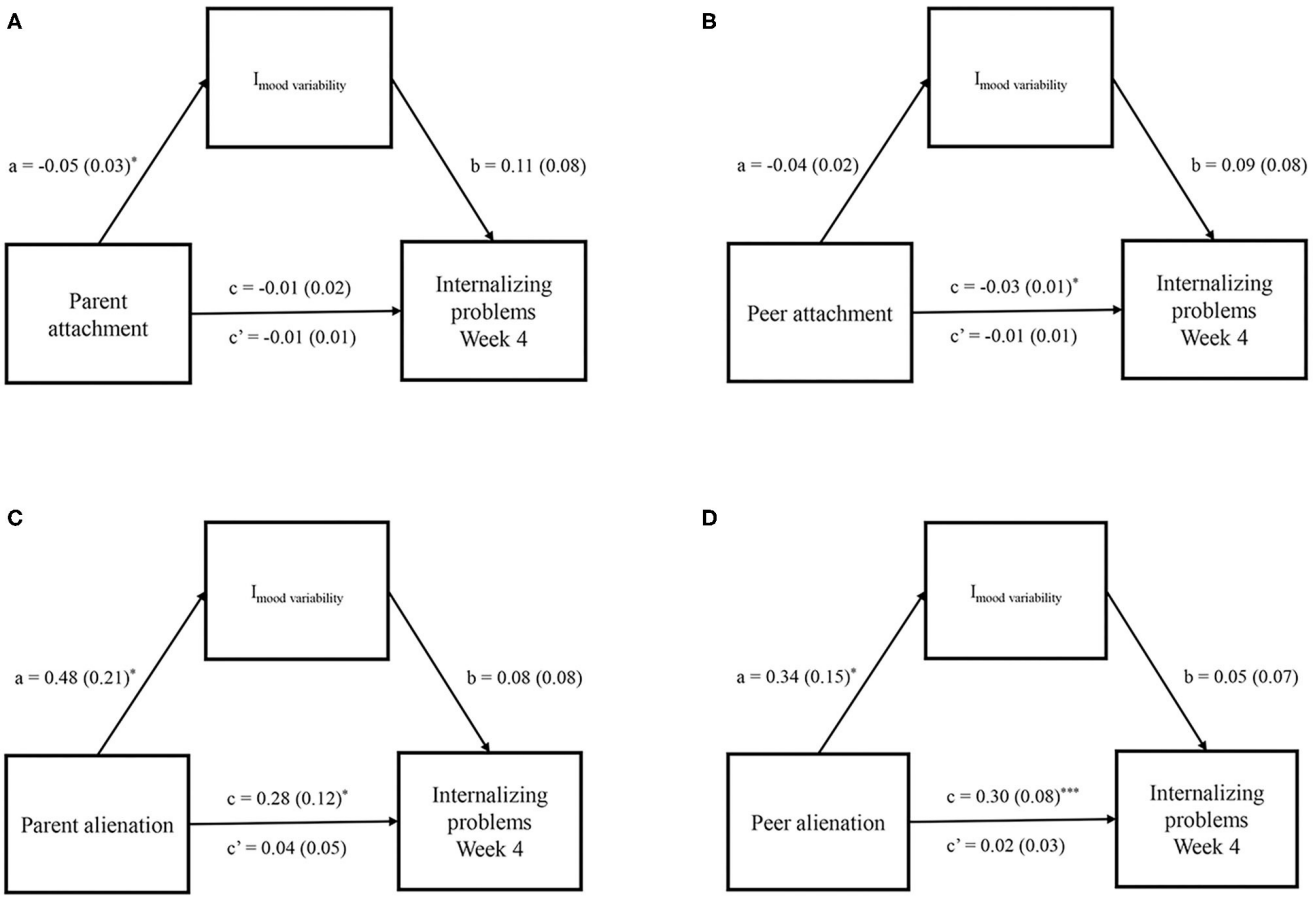
Adolescents' individual intercept op mood variability as mediator between parent attachment **(A)**, peer attachment **(B)**, parent alienation **(C)**, peer alienation **(D)**, and internalizing problems at the last assessment. *p <0.05 and ***p <0.001.

Our third aim was to examine whether attachment (peer or parent) was related to internalizing symptoms during the COVID-19 pandemic *via* mood variability. Results of the bootstrapped mediation analysis showed that there was no direct effect between parent attachment and internalizing problems (*B* = −0.01, *SE* = 0.02, *p* = 0.39) nor was this path mediated by the intercept of mood variability (Figure 3A, *B* = −0.01, *SE* = 0.01, 95% CI = [−0.03, 0.003], model R^2^ = 0.07). Interestingly, there was a direct effect between peer attachment and internalizing problems (*B* = −0.03, *SE* = 0.01, *p* = 0.026), yet this effect was not mediated by the intercept of mood variability (Figure 3B, *B* = −0.01, *SE* = 0.01, 95% CI = [−0.02, 0.01], model R^2^ = 0.12). This suggests that starting levels of mood variability did not mediate the association between parent or peer attachment and internalizing problems at week 4 of the study.

For the alienation subscale, a direct effect between parent alienation and internalizing problems was found (B = 0.28, *SE* = 0.12, *p* = 0.027) but the indirect effect of peer alienation on internalizing problems *via* mood variability was not significant (Figure 3C; *B* = 0.04, *SE* = 0.05, 95% CI = [−0.04, 0.16], model R^2^ = 0.13). Similarly, there was a direct effect of peer alienation on internalizing problems (*B* = 0.3. *SE* = 0.08, *p* = 0.001) but this was again not mediated by the intercept of mood variability (Figure 3D; *B* = 0.02, *SE* = 0.03, 95% CI = [−0.03, 0.10], model R^2^ = 0.26). Results did not change when sex was added as covariate (Supplementary Figure 1) or when the individual slope parameter (instead of the intercept parameter) of mood variability was included in the mediation model (Supplementary Figure 2). Thus, mood variability did not mediate the relation between experienced attachment and internalizing problems during the first few months of the COVID-19 pandemic.

### Time Spent With Peers

**Figure 4:**
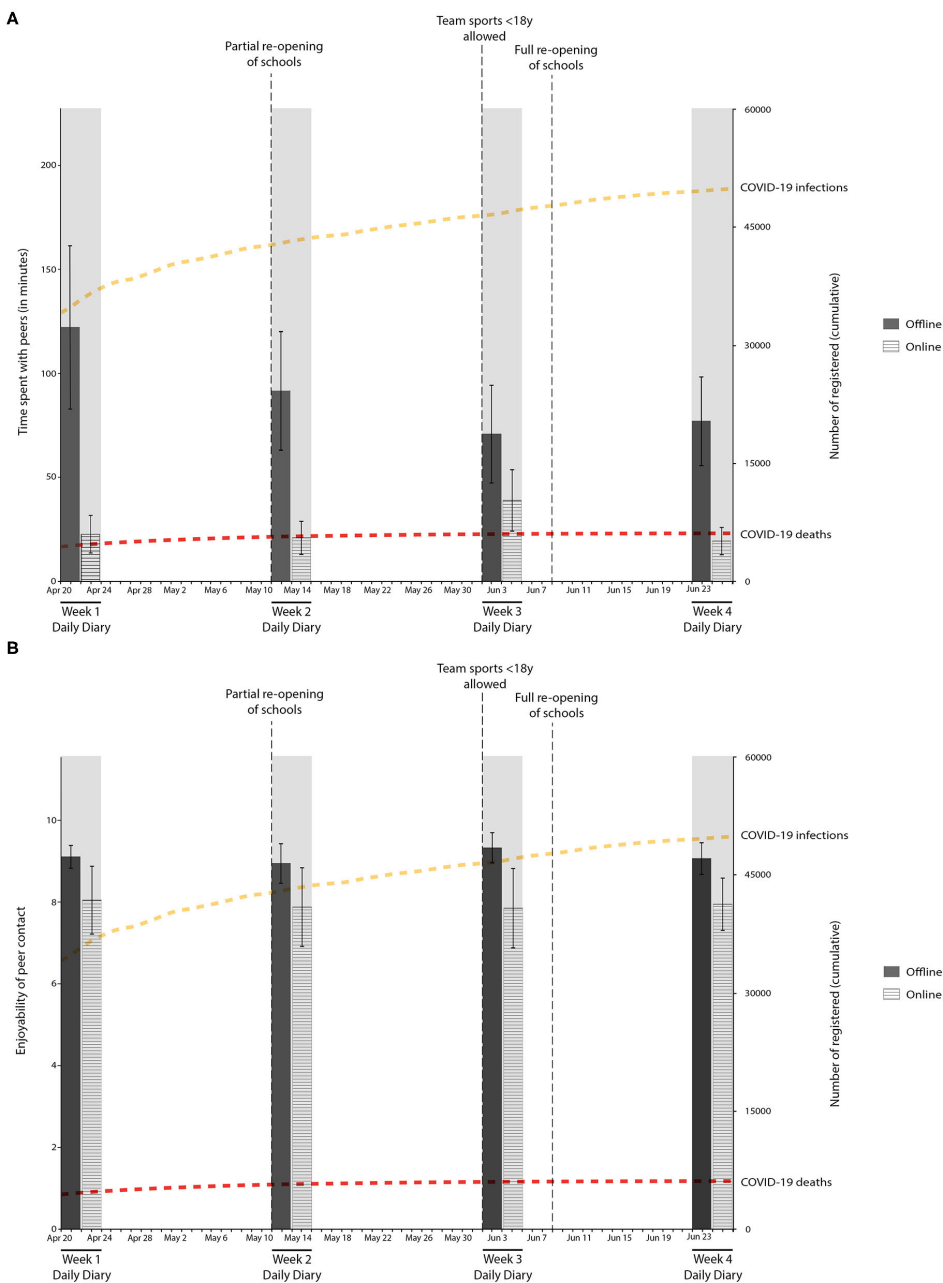
Average time **(A)** and grade **(B)** of offline and online peer contact during the first few months of the COVID-19 pandemic.

Last, we explored how time with peers was related to early adolescents' mood variability during different weeks of the COVID-19 pandemic. As shown in Table 3 and Figure 4, adolescents spent more time with their peers offline during the beginning of the COVID-19 pandemic which gradually declined across the weeks. Online time with peers remained relatively stable, with somewhat more time online during week 3. Thus, the decrease in offline contact was not compensated by increased online contact across the different assessment weeks. Regarding the enjoyability of the contact, adolescents rated the offline contact as highly enjoyable (*M* = 8.80–9.14). Online contact was also rated as highly enjoyable (*M* = 7.66–7.98) but significantly lower than offline contact (all *p*'s <0.001). Correlations of time and enjoyability of online and online peer contact between the different assessment weeks are shown in Supplementary Table 5.

**Table 3:** Means, standard deviations and correlations with mood variability of time and grade of offline and online peer contact during the different assessment weeks.

	**Week 1 (April 20–24, 2020)**			**Week 2 (May 11–15, 2020)**			**Week 3 (June 1–5, 2020)**			**Week 4 (June 22–26, 2020)**		
	***M***	***SD***	***r**_***mv***_*	***M***	***SD***	***r**_***mv***_*	***M***	***SD***	***r**_***mv***_*	***M***	***SD***	***r**_***mv***_*
**Offline**												
Time (minutes)	117.98	122.26	−0.20	89.69	90.49	−0.04	72.91	75.05	−0.11	77.93	68.86	−0.05
Enjoyability	9.08	0.75	−0.37^*^	8.80	1.03	−0.24	9.14	0.92	−0.36^*^	9.04	0.88	−0.24
**Online**												
Time (minutes)	23.04	28.27	0.03	19.82	24.08	−0.12	36.00	46.32	−0.16	21.04	25.70	−0.02
Enjoyability	7.72	1.82	−0.11	7.66	1.59	−0.19	7.87	1.72	−0.06	7.98	1.28	−0.01

*Enjoyability reflects the rating of how much the adolescent enjoyed the offline or online contact on a scale from 1 to 10. r_mv_ = Pearson's correlation coefficient between offline and online peer contact and mood variability per week.*

* *p < 0.05*.

To explore the effect of the social containment measures during the COVID-19 pandemic on early adolescents mood variability, mood variability was correlated with the average offline/online time spent with peers and average enjoyability of peer contact offline/online for each week separately. As shown in Table 3, a number of correlations were found between the enjoyability of offline contact and mood variability each week. Specifically, during week 1 (*r* = −0.37, *p* = 0.02) and week 3 (*r* = −0.36, *p* = 0.05) of the study, there was a significant negative correlation between mood variability and how enjoyable adolescents rated their offline contact, indicating lower mood variability with higher reported enjoyability. The correlation in week 1 (but not week 3) was also significant after accounting for sex (Supplementary Table 6). Our results provide preliminary evidence that early adolescents' mood variability was related to the quality of the offline contact with peers during the COVID-19 pandemic but not with the quality of online peer contact or the time they spent with their peers (either offline or online).

## Discussion

The impact of COVID-19 pandemic and the subsequent social containment measures on emotional well-being of adolescents is a topic of high concern all over the world. In the current study, we examined emotional well-being by examining the relation between mood variability, perceived attachment, and internalizing problems early adolescence (9–12 years) during 4 weeks (April 2020–June 2020) of different containment measures in the Netherlands. This study yielded four main findings. First, mood variability in early adolescents remained relatively stable across the first few months of the COVID-19 pandemic. Second, individual differences in levels of mood variability were related to the level of experienced attachment. Early adolescents who experienced low levels of attachment to their parents or peers, and specifically who felt a low connection to their parents or peers (i.e., high levels of alienation), reported more fluctuations in their day-to-day mood compared to early adolescents who experienced a strong level of attachment to their parents or peers. Third, experienced attachment to parents and peers was negatively related to internalizing problems. However, this association was not mediated by higher levels of mood variability. Fourth and last, higher quality of offline, but not online contact with peers was linked to adolescents' lower mood variability.

Our main finding of stable mood levels during the first few months of the COVID-19 pandemic in early adolescents aged 9–12 years was in contrast with our expectation of decreased mood variability with the easement of social containment measures. Interestingly, previous studies on mood levels during the first few months of the COVID-19 pandemic are mixed as studies showed both an increase in negative mood (Li et al., 2020; Orgilés et al., 2020; Whittle et al., 2020; De Quervain et al., 2020; Barendse et al., 2021; Luijten et al., 2021) as well as a stable or decrease in negative mood (Van De Groep et al., 2020; Achterberg et al., 2021; Fried et al., 2021) in children, adolescents, and college students. These seemingly discrepant findings might be explained by methodological differences, such as parent vs. child report. Moreover, differences in social containment measurements might relate to different psychological stressors such as re-organization of family life, social isolation, fear of death of relatives, economic and academic concerns (Fegert et al., 2020). More severe containment measure, such as social isolation, may induce more distress and therefore higher levels of mood variability. In the Netherlands, adolescents younger than 13 years old were still allowed to meet their peers without considering the social distance rules during the first acute phase of the pandemic, while adolescents older than 13 years could only meet their peers while keeping 1.5 m distance. In contrast, other European countries were in complete lockdown during the acute phase with no possibility for children and adolescents to meet their peers offline. This may have resulted in higher distress, social isolation, and changes in mood and well-being during the first few weeks or months of the pandemic (Orgilés et al., 2020), compared to adolescents in the Netherlands.

We also demonstrated that social connectedness, measured by experienced peer and parent attachment, was associated with individual differences in mood variability among early adolescents. That is, early adolescents who felt less alienated from their parents or peers experienced lower variability in mood during the COVID-19 pandemic, but not with changes within mood variability over time during the COVID-19 pandemic. This finding is in line with previous research showing that experienced levels of attachment is a protective factor against high levels of distress and may reduce fluctuations in mood (Laible et al., 2000; Wilkinson, 2004). So, resilience against mood fluctuations in stressful situations, such as the current COVID-19 pandemic, seems to be related to experienced parent and peer attachment during early adolescence. We further found that mood variability did not mediate the expected link between experienced parent or peer attachment and internalizing problems. It is possible that we did not find a mediation due to relatively low levels of internalizing problems and mood variability. Moreover, it has been suggested that heightened mood variability is a transition phase from a healthy mental state to a state of psychopathology (Houben et al., 2015). The duration of the study may have limited us from finding (1) changes in mood variability during this relative short-time window of 3 months, even though this was a period with strong contextual changes in social containment and (2) a mediation effect of mood variability in the relationship between attachment and internalizing problems, as the latter may develop at a later time with the prolonged effects of the pandemic and the subsequent social containment measurements. With the ongoing progression of the COVID-19 pandemic, future research should closely continue monitoring levels of mood of children and adolescents, and examine how this relates to their daily activities (i.e., school performance, school motivation, and social activities; Klootwijk et al., 2021). With the prolonged duration of the COVID-19 pandemic, the negative effects on emotional well-being of children and adolescents become more visible (Green et al., 2021; Weissman et al., 2021).

Last, we explored whether the time early adolescents spent with their peers online and offline during the COVID-19 pandemic was related to their mood variability. We focused on both offline and online experiences, given that social interaction not only occur face-to-face. Also, prior studies suggest that online media (e.g., WhatsApp, gaming) is also an important source for children and adolescents to interact with their peers and share some of the same characteristics as face-to-face interactions with friends, such as self-disclosure of personal information as well as emotional support (Yau and Reich, 2018; Boer et al., 2020; Rutledge, 2020). Our results show that even though both offline and online contact were rated as highly enjoyable, early adolescents favored offline contact. Moreover, ratings of enjoyability for only offline contact, but not how much time of offline contact, were linked to lower mood variability. This is in line with studies showing that specifically the quality of peer contact is an important predictor for psychological adjustment (Hussong, 2000; Waldrip et al., 2008). Moreover, participants were between 9 and 12 years old and therefore their social media use may be less frequent or intense than for adolescents older than 12 years (Craig et al., 2020). It is very likely that the in-depth relationship adolescents have with their peers online evolve across adolescence and therefore potentially also the relation with mood variability and mental well-being.

In line with prior studies, our study suggest that there are substantial individual differences how the first few months of the COVID-19 pandemic impacted the emotional well-being of children and adolescents (Caffo et al., 2020; Fegert et al., 2020; Whittle et al., 2020). Our study showed that not all children and adolescents are negatively affected by the pandemic and some children and adolescents may even benefit from these societal changes by creating opportunities for more emotional stability (Bruining et al., 2021). For example, forced familial proximity and the elimination of school-related stressors such as bullying, peer pressure or academic stress (Timmons and Margolin, 2015; Achterberg et al., 2021) may actually have relieved some children, adolescents, and their families from daily stressors and struggles with work-family balance. Moreover, strong connectedness and the quality of contact with parents and peers seems to facilitate resilience to distress and daily mood fluctuation in children and adolescents. Whereas, feeling socially disconnected during the pandemic is associated with higher levels of anxiety and depressive symptoms (Magson et al., 2020). As noted by prior studies (e.g., Bruining et al., 2021), the COVID-19 pandemic changes our complete society and it is important to look also at the opportunities provided by this pandemic. For example, reducing academic stress and family stress might prevent high fluctuations in daily mood and mental health problems in young adolescents. As such, the societal changes enforced by the COVID-19 pandemic may provide fruitful lessons that could improve the lives and emotional well-being of children and adolescents in the future.

This study has several strengths, such as assessing daily diaries over different weeks during different social containment measures in the Netherlands, focusing on mood variability and exploratory assessed quantity and quality of peer interactions. Nevertheless, there are also some limitations that need to be considered. First, although participants were sampled from an ongoing longitudinal study, no information on mood variability and internalizing problems prior to the COVID-19 pandemic were available. Therefore, it is not clear whether the found individual differences in mood variability are a consequence of the COVID-19 pandemic and subsequent containment measures or whether these are stable individual differences in mood variability. Moreover, the associations between attachment and mood variability are correlational, and no causal effects can be inferred. Second, our sample was comprised of typically developing early adolescents from a high socioeconomic background. It is likely that the psychological impact of the COVID-19 pandemic is higher for adolescents from less economic beneficial environments (Green et al., 2021) or who experienced psychiatric problems before (Zijlmans et al., 2021). Additionally, our sample consisted of typically intelligent and gifted early adolescents. We did not find any differences between typically intelligent and gifted early adolescents on mood variability, perceived attachment or internalizing problems during the COVID-19 pandemic. These findings are in line with studies showing no or small differences between gifted and typically intelligent students in psychological well-being (Zeidner and Shani-Zinovich, 2011; Kroesbergen et al., 2016; Bergold et al., 2020). However, due to the small size of our subsamples, we need to be cautious in interpreting our findings. It would, however, be of great interest for future research to examine how gifted students cope with the COVID-19 pandemic, given that some studies suggest that gifted students are more likely to experience anxiety and depressive symptoms due to higher sensitivity (Karpinski et al., 2018; Armstrong et al., 2019). Last, the reliability of the alienation subscale of parent attachment was low (ω = 0.34). Nevertheless, the found link between parent alienation and mood variability resembles that of peer alienation (which had a reliability of ω = 0.73). This resemblance strengthens our finding that attachment, and specifically alienation, may relate to early adolescents' mood variability.

### Conclusion

The vaccination programs have largely started and show their positive effects, yet the COVID-19 pandemic (or future health pandemics) is likely to influence our lives for a longer period. The potential psychological stress associated with the COVID-19 pandemic and the slow return to normality may be more chronic and may specifically impact young people that need to ascertain their independence in the upcoming years. Importantly, our study highlight that connectedness to parents and peers may protect against mood fluctuations. This is an important insight for policy makers to consider when new containment measures need to be made as constraining peer interactions may take away (early) adolescents from crucial input during this developmental phase of their lives.

## Data Availability Statement

The analysis scripts are available on our OSF-page (https://osf.io/3fqky/) and data is available at dataverse (link can be found on our OSF-page https://osf.io/3fqky/).

## Ethics Statement

The studies involving human participants were reviewed and approved by Commissie Ethiek Psychologie, Leiden University; Nr2354. Written informed consent to participate in this study was provided by the participants' legal guardian/next of kin.

## Author Contributions

JA, MB, and KZ contributed to conception and design of the study. JA organized the data and performed the statistical analyses supervised by MB and KZ. Visualization was done by JA and MB. JA and MB prepared the original draft. All authors contributed to manuscript revision, read, and approved the submitted version.

## Conflict of Interest

The authors declare that the research was conducted in the absence of any commercial or financial relationships that could be construed as a potential conflict of interest.

## Publisher's Note

All claims expressed in this article are solely those of the authors and do not necessarily represent those of their affiliated organizations, or those of the publisher, the editors and the reviewers. Any product that may be evaluated in this article, or claim that may be made by its manufacturer, is not guaranteed or endorsed by the publisher.

## Supplementary Material

Supplementary Figure 1 Adolescents' individual intercept of mood variability as mediator between parent attachment **(A)**, peer attachment **(B)**, parent alienation **(C)**, peer alienation **(D)**, and internalizing problems at the last assessment, controlled for sex.

Supplementary Figure 2 Adolescents' individual slope of mood variability as mediator between parent attachment **(A)**, peer attachment **(B)**, parent alienation **(C)**, peer alienation **(D)**, and internalizing problems at the last assessment.

Supplementary Table 1 Means (M) and Standard Deviations (SD) of the Mood Scales as well as the Average Negative Mood Across the Four Subscales.

Supplementary Table 2 Factor Loadings of the Different Mood Variability Scales.

Supplementary Table 3 Omega Coefficient (ω) for the Overall and Subscales of the Items of the Inventory of Parent and Peer Attachment.

Supplementary Table 4 Regression Analysis of Parent and Peer Attachment Scales on the Slope Parameters of Mood Variability.

Supplementary Table 5 Correlations of Time and Grade Spent with Peers Offline and Online and Peer Attachment.

Supplementary Table 6 Correlation Coefficient with Mood Variability per Week Controlled for Sex.
