# Supplementary figures and images for: Mood Variability Among Early Adolescents in Times of Social Constraints: A Daily Diary Study During the COVID-19 Pandemic
Source: Front Psychol. 2021 Aug 24;12:722494. doi: 10.3389/fpsyg.2021.722494 (PMC8421763; doi:10.3389/fpsyg.2021.722494)

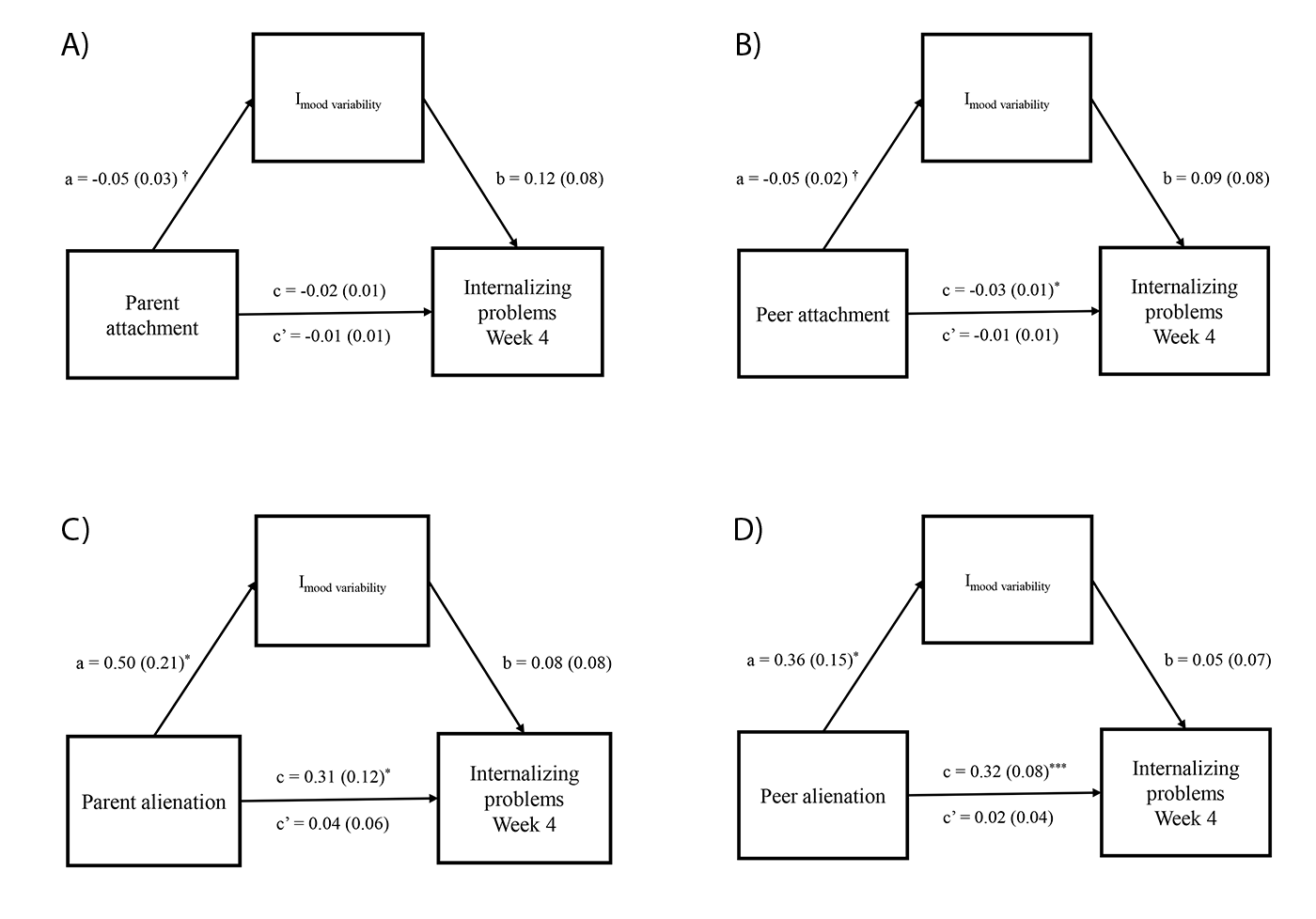

Supplement: Supplementary Figure 1 — Adolescents' individual intercept of mood variability as mediator between parent attachment (A), peer attachment (B), parent alienation (C), peer alienation (D), and internalizing problems at the last assessment, controlled for sex. [file Image_1.tif]

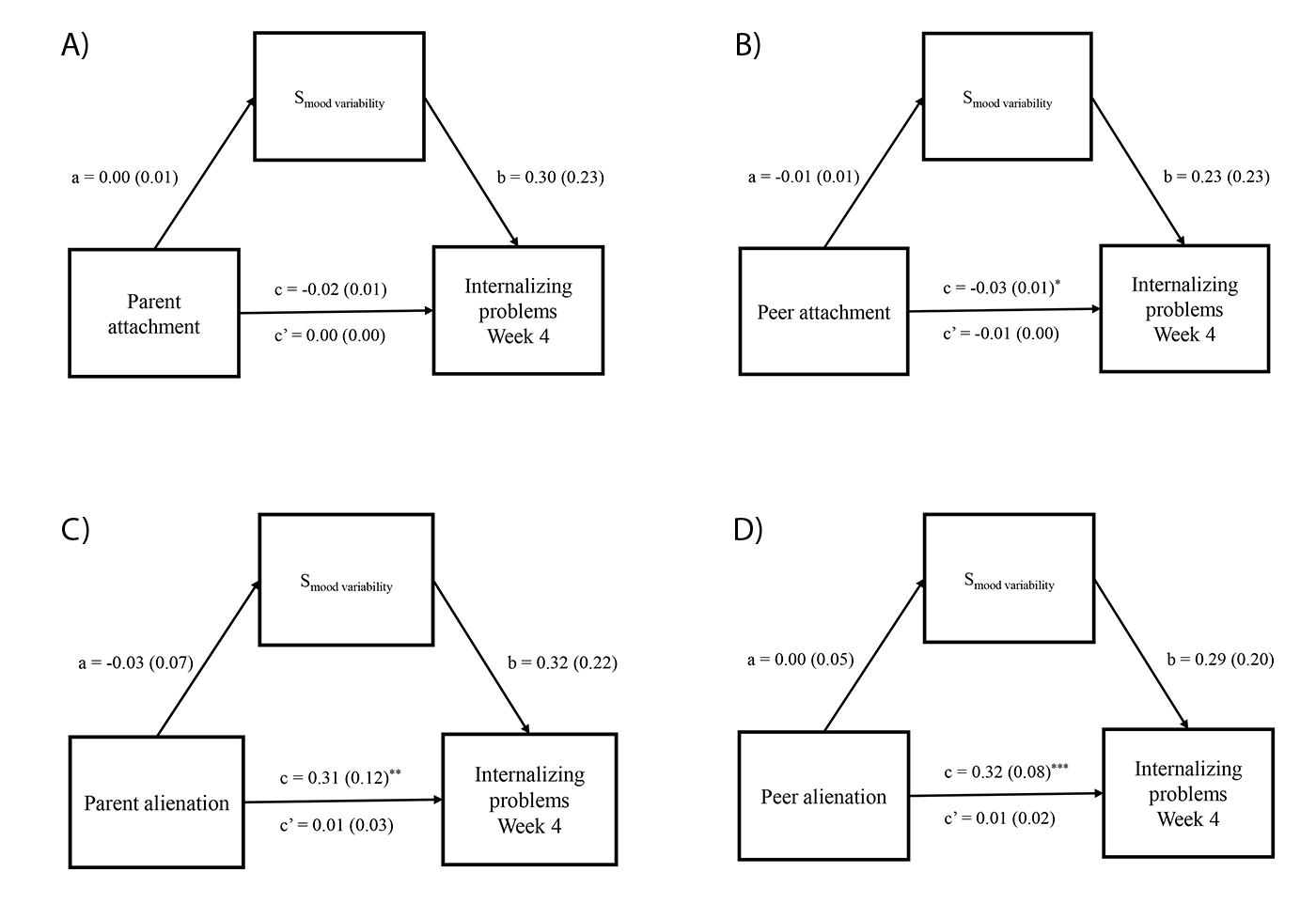

Supplement: Supplementary Figure 2 — Adolescents' individual slope of mood variability as mediator between parent attachment (A), peer attachment (B), parent alienation (C), peer alienation (D), and internalizing problems at the last assessment. [file Image_2.tif]
