# Supplementary material for: Mood Variability Among Early Adolescents in Times of Social Constraints: A Daily Diary Study During the COVID-19 Pandemic
Source: Front Psychol. 2021 Aug 24;12:722494. doi: 10.3389/fpsyg.2021.722494 (PMC8421763; doi:10.3389/fpsyg.2021.722494)
Supplement: Supplementary Table 1 — Means (M) and Standard Deviations (SD) of the Mood Scales as well as the Average Negative Mood Across the Four Subscales. [file Data_Sheet_1.PDF]

S1 Table. Means (*M*) and Standard Deviations (*SD*) of the Mood Scales as well as the Average Negative Mood Across the Four Subscales.

	Week 1 (April 20-24)		Week 2 (May 11-15)		Week 3 (June 1-5)		Week 4 (June 22-26)	
	M	*SD*	*M*	*SD*	*M*	*SD*	*M*	*SD*
Happiness	7.07	1.19	7.02	1.46	7.28	1.12	7.20	1.06
Anger	1.56	0.68	1.54	0.80	1.54	0.76	1.43	0.63
Sadness	1.44	0.72	1.50	0.89	1.41	0.58	1.40	0.53
Anxiety	1.43	0.71	1.29	0.50	1.23	0.32	1.32	0.53
Negative mood[1]	1.84	0.61	1.83	0.69	1.73	0.52	1.74	0.46

[1] Negative mood reflects the average of the four mood scales per week. To this end, happiness was reverse coded.

S2 Table. Factor Loadings of the Different Mood Variability Scales.

	Week 1 (April 20-24)		Week 2 (May 11-15)	Week 3 (June 1-5)	Week 4 (June 22-26)
	F.1	F.2	F.1	F. 1	F. 1
Variability					
Happiness	.54	**-.63**	.85	.79	.50
Anger	**.82**		.84	.72	.73
Sadness	**.88**		.84	.78	.77
Anxiety	.40	**.76**	.55	.63	.80
Eigenvalue	1.90	47.43	2.44	2.15	2.01
Variance in %	1.05	26.20	60.88	53.85	50.18

Note. F = Factor extracted according to factor analysis. Bold factor loadings indicate preferred factor.

S3 Table. Omega Coefficient (ω) for the Overall and Subscales of the Items of the Inventory of Parent and Peer Attachment.

	Mother<br>ω	Father<br>ω	Peer<br>ω
Overall	.70	.80	.87
Alienation	.34	.51	.73
Communication	.61	.76	.84
Trust	.79	.83	.75

S4 Table. Regression Analysis of Parent and Peer Attachment Scales on the Slope Parameters of Mood Variability.

	Overall	Alienation	Communication	Trust
	B (SE)	*B (SE)*	*B (SE)*	*B (SE)*
**Parent attachment**				
Step 1				
Constant	0.00 (0.11)	0.03 (0.07)	0.01 (0.08)	0.03 (0.15)
Attachment	0.00 (0.01)	-0.02 (0.07)	0.00 (0.01)	-0.00 (0.01)
Step 2				
Constant	0.22 (0.12) †	0.31 (0.11) **	0.22 (0.10) *	0.31 (0.16) †
Attachment	0.00 (0.01)	-0.05 (0.07)	0.00 (0.01)	0.00 (0.01)
Sex	-0.15 (0.05) **	-0.15 (0.05) **	-0.16 (0.05) **	-0.15 (0.05) **
**Peer attachment**				
Step 1				
Constant	0.06 (0.07)	0.02 (0.04)	0.04 (0.04)	0.08 (0.08)
Attachment	-0.01 (0.01)	-0.01 (0.05)	-0.01 (0.01)	-0.01 (0.01)
Step 2				
Constant	0.25 (0.09) **	0.28 (0.09) **	0.26 (0.08) **	0.27 (0.09) **
Attachment	0.00 (0.01)	-0.02 (0.05)	0.00 (0.01)	0.00 (0.01)
Sex	-0.15 (0.05) **	-0.15 (0.05) **	-0.15 (0.05) **	-0.15 (0.05) **

Note. Analyses were performed with the transformed data. For the overall attachment, communication and trust subscale a square transformation and for the alienation subscale a square root transformation was used.

$^{†}p < .10$, $^{*}p < .05$, $^{**}p <. 01$, $^{***}p < .001$.

S5 Table. Correlations of Time and Grade Spent with Peers Offline and Online and Peer Attachment

		1	2	3	4	5	6	7	8	9	10	11	12	13	14	15	16	17	18	19
1	Time offline T1	-																		
2	Time offline T2	.17	-																	
3	Time offline T3	.09	**.47**	-																
4	Time offline T4	.08	**.34**	**.33**	-															
5	Enjoyability offline T1	-.06	.06	-.01	-.30	-														
6	Enjoyability offline T2	-.09	.27	.16	-.01	**.40**	-													
7	Enjoyability offline T3	.02	-.33	-.16	-.24	.17	.26	-												
8	Enjoyability offline T4	.00	-.15	**-.37**	-.13	-.08	.04	**.38**	-											
9	Time online T1	.08	.03	-.03	-.04	.06	.27	**.34**	.07	-										
10	Time online T2	.14	.20	.01	.00	.23	.18	.21	.06	**.62**	-									
11	Time online T3	-.01	.21	.12	-.09	.15	.14	.07	.05	**.29**	**.58**	-								
12	Time online T4	.25	.17	.08	.02	-.24	.07	.14	-.12	**.63**	**.41**	**.60**	-							
13	Enjoyability online T1	-.18	-.02	-.18	.03	.16	**.39**	**.41**	.37	**.33**	.25	.15	.11	-						
14	Enjoyability online T2	.01	-.02	.01	.11	.19	**.32**	**.41**	.06	**.29**	**.37**	-.04	-.02	**.51**	-					
15	Enjoyability online T3	-.17	-.13	-.28	-.20	.26	**.45**	**.64**	**.39**	.30	.29	.30	.11	**.73**	**.51**	-				
16	Enjoyability online T4	.18	-.20	-.32	-.09	-.07	.03	**.51**	**.39**	.22	.28	.27	.25	**.60**	**.42**	.78	-			
17	Peer attachment T1	.15	.06	.08	-.01	.13	.16	**.37**	**.41**	.09	.14	-.27	.12	.18	.13	.02	-.01	-		
18	Peer alienation T1	-.21	-.11	-.14	-.02	-.09	-.04	-.27	-.18	-.07	-.11	.23	-.09	-.02	-.19	.08	-.04	**-.80**	-	
19	Peer communication T1	.05	-.03	-.02	-.02	.18	.20	.31	.32	.05	.17	-.10	.16	.23	.09	.12	-.01	**.78**	**-.32**	-
20	Peer trust T1	.14	.09	.10	-.01	-.01	.11	.28	**.49**	.10	.05	**-.38**	.06	.16	.06	-.01	-.05	**.91**	**-.72**	**.57**

Note. Correlation coefficients in bold are significant at $p < .05$.

S6 Table. Correlation Coefficient with Mood Variability per Week Controlled for Sex.

	Week 1 (April 20-24)	Week 2 (May 11-15)	Week 3 (June 1-5)	Week 4 (June 22-26)
Time offline	-.17	-.04	-.04	-.06
Enjoyability offline	-.38*	-.23	-.32	-.24
Time online	.08	-.14	-.19	-.01
Enjoyability online	-.13	-.19	-.10	.00

* $p < .05$
